# Measuring the Complexity of Consciousness

**DOI:** 10.3389/fnins.2018.00424

**Published:** 2018-06-27

**Authors:** Xerxes D. Arsiwalla, Paul Verschure

**Affiliations:** ^1^Institute for Bioengineering of Catalunya, Barcelona, Spain; ^2^Barcelona Institute for Science and Technology, Barcelona, Spain; ^3^Universitat Pompeu Fabra, Barcelona, Spain; ^4^Institució Catalana de Recerca i Estudis Avançats, Barcelona, Spain

**Keywords:** consciousness in the clinic, computational neuroscience, complexity measures, information theory, clinical scales

## Abstract

The grand quest for a scientific understanding of consciousness has given rise to many new theoretical and empirical paradigms for investigating the phenomenology of consciousness as well as clinical disorders associated to it. A major challenge in this field is to formalize computational measures that can reliably quantify global brain states from data. In particular, information-theoretic complexity measures such as integrated information have been proposed as measures of conscious awareness. This suggests a new framework to quantitatively classify states of consciousness. However, it has proven increasingly difficult to apply these complexity measures to realistic brain networks. In part, this is due to high computational costs incurred when implementing these measures on realistically large network dimensions. Nonetheless, complexity measures for quantifying states of consciousness are important for assisting clinical diagnosis and therapy. This article is meant to serve as a lookup table of measures of consciousness, with particular emphasis on clinical applicability. We consider both, principle-based complexity measures as well as empirical measures tested on patients. We address challenges facing these measures with regard to realistic brain networks, and where necessary, suggest possible resolutions.

## 1. Introduction

In patients with disorders of consciousness (DoC), such as locked-in syndrome, coma or the vegetative state, levels of consciousness are clinically assessed through both, behavioral tests as well as neurophysiological recordings. In particular, these methods are used to assess levels of wakefulness (or arousal) and awareness (Laureys et al., 2004; Laureys, 2005). These observations have culminated in a two dimensional operational definition of clinical consciousness. Assessments of awareness use behavioral and/or neurophysiological (fMRI or EEG) protocols to gauge performance on cognitive tasks (Fingelkurts et al., 2012; De Pasquale et al., 2015). Assessments of wakefulness are based on metabolic markers (when reporting is not possible) such as estimates of glucose uptake in the brain, calibrated from positron emission tomography (PET) imaging (Bodart et al., 2017). A clinical definition of consciousness is useful when one wants to classify closely associated states and disorders of consciousness. For instance, in identifying clusters of related disorders on a bivariate scale, where wakefulness and awareness label orthogonal axes. Under healthy conditions, these two levels or scales are almost linearly correlated, as in conscious wakefulness (which shows high levels of arousal accompanying high levels of awareness), or in various stages of sleep (displaying low arousal with low awareness in deep sleep and slightly increased awareness during dreams). However, in pathological states, wakefulness without awareness can be observed in the vegetative state (Laureys et al., 2004), while transiently reduced awareness is observed following seizures (Blumenfeld, 2012). Patients in the minimally conscious state show intermittent and limited non-reflexive and purposeful behavior (Giacino et al., 2002; Giacino, 2004), whereas patients with hemi-spatial neglect display reduced awareness of stimuli contralateral to the side where brain damage has occurred (Parton et al., 2004). Nevertheless, while awareness is directly associated to consciousness, the role of wakefulness is less direct. Of course, in all organic life forms metabolic processes are necessary precursors of consciousness, if at all it exists in those organisms. Still it is not clear whether wakefulness does more than serving merely as a necessary condition for biological consciousness (Fingelkurts et al., 2014). Also it is not known if states of consciousness can exist, which do not depend on any form of wakefulness.

Given the above proposals for classifying states and disorders of consciousness, the topic of this article will concern complexity measures that describe awareness. The question is how can one reliably quantify states of awareness from patient data? This is particularly important for non-communicative patients as in coma or minimal consciousness (MC). It is for this reason that a variety of dynamical complexity measures have been developed for consciousness research. In this article, we first describe theoretically-grounded complexity measures and the challenges one faces when applying these measures to realistic brain data. We then outline alternative empirical approaches to classify states and disorders of consciousness. We end with a discussion on how these two approaches might inform each other.

## 2. Measures of integrated information

Dynamical complexity measures are designed to capture both, the network's topology as well as its causal dynamics. The most prominent of these measures is integrated information (also known as Φ), which was introduced in Tononi et al. (1994). Φ is defined as the quantity of information generated by a system as a whole, over and above that of its parts, taking into account the system's causal dynamical interactions. This reflects the intuition going back to William James that conscious states are integrated, yet diverse. Φ seeks to formalize this intuition in terms of the system's complexity. It is believed that complexity stems from simultaneous integration and differentiation of information generated by the system's collective states. Here differentiation refers to local functional specialization, while integration, as a complementary design principle, results in global or distributed coordination within the system. It is this interplay that generates integrated yet diversified information, which is believed to support cognitive and behavioral states in conscious agents. Early proposals formalizing integrated information can be found in Tononi et al. (1994), Tononi and Sporns (2003), and Tononi (2004). More recently, considerable progress has been made toward the development of a normative theory and applications of integrated information (Balduzzi and Tononi, 2008; Barrett and Seth, 2011; Tononi, 2012; Arsiwalla and Verschure, 2013; Oizumi et al., 2014; Arsiwalla and Verschure, 2016a,c; Krohn and Ostwald, 2016; Tegmark, 2016; Arsiwalla et al., 2017b; Arsiwalla and Verschure, 2017). As a whole vs. parts measure, integrated information has been defined in several different ways. Some examples are neural complexity (Tononi et al., 1994), causal density (Seth, 2005), Φ from integrated information theory: IIT 1.0, 2.0 & 3.0 (Tononi, 2004; Balduzzi and Tononi, 2008; Oizumi et al., 2014), stochastic interaction (Wennekers and Ay, 2005; Ay, 2015), stochastic integrated information (Barrett and Seth, 2011; Arsiwalla and Verschure, 2013, 2016b) and synergistic Φ (Griffith and Koch, 2014; Griffith, 2014). In Table 1 we summarize these theoretical measures along with their associated information metrics.

**Table 1:** A list of theoretical complexity measures and their respective information metrics.

**Integrated information measures**	**Information metrics**
Neural complexity	Mutual information (MI)
Causal density	Granger causality (GC)
Stochastic interaction	Kullback-Leibler divergence (KLD)
IIT 1.0 & 2.0	KLD
Stochastic integrated information	MI or KLD
IIT 3.0	Earth mover's distance
Synergistic Φ	Synergistic information

However, computing integrated information for large neurophysiological datasets has been challenging due to both, computational difficulties and limits on domains where these measures can be implemented. For instance, many of these measures use the minimum information partition of the network. This involves evaluating a large number of network configurations (more precisely, Bell number of network configurations), which makes their computational cost extremely high for networks of large size. As for domains of applicability, the measure of Balduzzi and Tononi (2008) has been formulated for deterministic, discrete-state, Markovian systems with the maximum entropy distribution. On the other hand, the measure of Barrett and Seth (2011) has been designed for stochastic, continuous-state, non-Markovian systems. The latter also admits dynamics with any empirical distribution (though in practice, it is easier to use with Gaussian distributions). The definition in Barrett and Seth (2011) is based on mutual information, whereas Balduzzi and Tononi (2008) uses an integrated information measure based on Kullback-Leibler divergence. However, it has also been noted that in some cases the measure of Barrett and Seth (2011) gives negative values, which complicates its interpretation. In contrast, the Kullback-Leibler based measure of Balduzzi and Tononi (2008) computes the information generated during state transitions and remains positive in the regime of stable dynamics. This lends it a natural interpretation as an integrated information measure. Note that both measures (Balduzzi and Tononi, 2008; Barrett and Seth, 2011) use a normalization scheme in their calculations. This normalization inadvertently introduces ambiguities in computations. Normalization is used for the purpose of determining the network partition that minimizes integrated information. However, it turns out that a normalization dependent choice of partition ends up affecting the computed value of Φ, thereby introducing ambiguity. An alternate measure based on “Earth-Mover's distance” was proposed in Oizumi et al. (2014) for discrete-state systems. This does away with normalization. However, this formulation lies outside the scope of standard information theory and is challenging for running computations on large networks.

More recently, the above-mentioned issues including normalization and scaling of computational costs with network size have been thoroughly addressed in Arsiwalla and Verschure (2016b). This study developed a formulation of stochastic integrated information based on the Kullback-Leibler divergence between the conditional multivariate distribution on the set of network states vs. the corresponding factorized distribution over its parts. The corresponding measure makes use of the maximum information partition rather than the minimum information partition used in previous measures. Using this formulation, Φ can be computed for large-scale networks with linear stochastic dynamics, for stationary (attractor) as well as non-stationary states (Arsiwalla and Verschure, 2016b). This work also demonstrated the first computation of integrated information for resting-state data of the human brain connectome. The connectome network is reconstructed from cortical white matter tractography. The data used for this computation consists of 998 voxels with approximately 28,000 weighted symmetric connections (Hagmann et al., 2008). Arsiwalla and Verschure (2016b) show that the topology and dynamics of the healthy human brain in the resting-state generates greater information complexity than a weight-preserving random rewiring of the same network (for network dynamics of the connectome refer to Arsiwalla et al., 2013; Betella et al., 2014; Arsiwalla et al., 2015a,b). While this formulation of integrated information indeed works for very large networks, it is still limited to linearized dynamics. Of course this works well in the vicinity attractors, as in the resting-state. However, it would be desirable to extend this formulation to include non-linearities existing in brain dynamics.

## 3. Empirical measures

Ideally, integrated information was intended as a measure of awareness, one that could account for informational differences between states and also disorders of consciousness. However, as described above, for realistic brain dynamics and physiological data that task has in fact proven difficult. On the other hand, the basic conceptualization of consciousness in terms of integration and differentiation of causal information has motivated several empirical measures that seek to classify consciousness-related disorders from patient data. For example, Barrett et al. (2012) investigated changes in conscious levels using Granger Causality (GC) as a causal connectivity measure. Given two stationary time-series signals, Granger Causality measures “the extent to which the past of one assists in predicting the future of the other, over and above the extent to which the past of the latter already predicts its own future” (Granger, 1969), thus quantifying causal relations between two signaling sources. This was tested using electroencephalographic (EEG) data from subjects undergoing propofol-induced anesthesia. Recordings were made with source-localized signals at the anterior and posterior cingulate cortices. There Barrett et al. (2012) found “a significant increases in bi-directional GC in most subjects during loss of consciousness, especially in the beta and gamma frequency ranges.”

Other measures of causal connectivity include permutation entropy (Olofsen et al., 2008), transfer entropy (which extends Granger causality to the non-Gaussian case) (Wibral et al., 2014), symbolic transfer entropy (Staniek and Lehnertz, 2009) and permutation conditional mutual information (Li and Ouyang, 2010). Among these, permutation entropy has been tested on EEG data from patients administered with sevoflurane and propofol anesthesia. This measure has been successful in tracking anesthetic related changes in EEG patterns such as loss of high frequencies, spindle-like waves and delta waves. The other causal connectivity measures in this list have so far been applied to data from neuronal cultures or psychophysical paradigms. Many of them have shown results superior to Granger causality in the above-mentioned works. This suggests that they might be promising candidates as empirical consciousness measures.

An important measure that has been used as a clinical classifier of conscious states is the Perturbational Complexity Index (PCI), which was introduced by Casali et al. (2013) and tested on TMS-evoked potentials measured with EEG. PCI is calculated “by perturbing the cortex with transcranial magnetic stimulation (TMS) in order to engage distributed interactions in the brain and then compressing the resulting spatiotemporal EEG responses to measure their algorithmic complexity, based on the Lempel-Ziv compression.” For a given segment of EEG data, the Lempel-Ziv algorithm quantifies complexity by counting the number of distinct patterns present in the data. For instance, this can be proportional to the size of a computer file after applying a data compression algorithm. Computing the Lempel-Ziv compressibility requires binarizing the time-series data, based either on event-related potentials or with respect to a given threshold. Using PCI, Casali et al. (2013) were able to discriminate levels of consciousness during wakefulness, sleep, and anesthesia, as well as in patients who had emerged from coma and recovered a minimal level of consciousness. Later, the Lempel-Ziv complexity was also used by Schartner et al. (2015) on spontaneous high-density EEG data recorded from subjects undergoing propofol-induced anesthesia. Once again, a robust decline in complexity was observed during anesthesia. These are complexity measures based on data compression algorithms. A qualitative comparison between a data compression measure inspired by PCI and Φ was made in Virmani and Nagaraj (2016). While compression-based measures do seem to capture certain aspects of Φ, the exact relationship between the two is not completely clear. Nonetheless, these empirical measures have been useful for clinical purposes, in terms of broadly discriminating disorders of consciousness.

Another relevant complexity measure is the weighted symbolic mutual information (wSMI), introduced by King et al. (2013). This is a measure of global information sharing across brain areas. It evaluates “the extent to which two EEG channels present nonrandom joint fluctuations, suggesting that they share common sources.” This is done by first transforming continuous signals into discrete symbols, and subsequently computing the joint probabilities of symbol pairs between two EEG channels. Before computing the symbolic mutual information between two time-series signals, a weighting of symbols is introduced to disregard conjunctions of either identical or opposite-sign symbols from the two signal trains, as those cases could potentially arise from common source artifacts. In King et al. (2013) wSMI was estimated for 181 EEG recordings from awake but noncommunicating patients diagnosed in various disorders of consciousness (including 143 from patients in vegetative and minimally conscious states). This measure of information sharing was found to systematically increases with consciousness. In particular, it was able to distinguish patients in the vegetative state, minimally conscious state, and fully conscious state. In Table 2 we summarize empirical complexity measures commonly used in consciousness research along with their domains of application.

**Table 2:** A list of empirical complexity measures alongside their tested domains of application.

**Empirical measures**	**Tested application domains**
Granger causality	Wakefulness vs. propofol-induced anesthesia using EEG
Permutation entropy	Sevoflurane and propofol anesthesia using EEG
Perturbational complexity index	Wakefulness, sleep, anesthesia, coma and minimal consciousness using TMS-evoked EEG
Lempel-Ziv complexity	Wakefulness vs. propofol-induced anesthesia using EEG
Weighted symbolic mutual information	Vegetative, minimally conscious and fully conscious states using EEG

## 4. Discussion

The paradigm-shifting proposal that consciousness might be measurable in terms of information generated as a whole, over the sum of its parts, by the causal dynamics of the brain, has led to precise quantitative formulations of information-theoretic complexity measures. These complexity measures seek to operationalize the intuition that the complexity associated to consciousness arises from simultaneous integration and differentiation of the brain's structural and dynamical hierarchies. However, progress in this direction has faced practical challenges such as high computational cost upon scaling with network size. This is especially true with regard to realistic neuroimaging or physiological datasets. Even in the approach of Arsiwalla and Verschure (2016b), where both, the scaling and normalization problem have been solved, the formulation is still applicable only to linear dynamical systems. A possible way to extend this formulation to non-linear systems such as the brain might be to first solve the Fokker-Planck equations for these systems (as probability distributions will no longer remain Gaussian) and subsequently estimate entropies and conditional entropies numerically to compute Φ. Another solution to the problem might be to construct statistical estimators for the covariance matrices from data and then compute Φ.

In the meanwhile, at least for clinical purposes, it has been useful to have empirical complexity measures. These work as classifiers which very broadly discriminate between states of consciousness, such as wakefulness and anesthesia or between generic disorders of consciousness. However, these measures do not strictly correspond to integrated information. Some of them are based on signal compression, which does capture differentiation, though not directly integration. So far these methods have been applied on the scale of EEG datasets. One has yet to demonstrate their computational feasibility for larger datasets (which might only be a matter of time though). All in all, bottom-up approaches suggest important features that might help inform or constrain implementations of principle-based approaches. However, the latter are indispensable for ultimately understanding causal aspects of information generation and flow in the brain.

This article is intended as a lookup table spanning the landscape of both, theoretically-motivated as well as empirically-based complexity measures used in current consciousness research. Even though, for the purpose of this article, we have treated complexity as a global correlate of consciousness, there are indications that multiple complexity types, based on cognitive and behavioral control, might be important for a more precise classification of various states of consciousness (Arsiwalla et al., 2016b, 2017c; Bayne et al., 2016). This latter observation alludes to the need for an integrative systems approach to consciousness research (Fingelkurts et al., 2009; Arsiwalla et al., 2017a), especially one that is grounded in cognitive architectures and helps understand control mechanisms underlying systems level neural information processing (Arsiwalla et al., 2016a; Moulin-Frier et al., 2017).

## Author contributions

Both XA and PV contributed to the design, analysis, interpretation and writing of the manuscript.

### Conflict of interest statement

The authors declare that the research was conducted in the absence of any commercial or financial relationships that could be construed as a potential conflict of interest.
